# NMDAR Hypofunction Animal Models of Schizophrenia

**DOI:** 10.3389/fnmol.2019.00185

**Published:** 2019-07-31

**Authors:** Gloria Lee, Yi Zhou

**Affiliations:** Department of Biomedical Sciences, Florida State University College of Medicine, Tallahassee, FL, United States

**Keywords:** NMDAR, NMDAR hypofunction, NMDAR antagonists, knockout mice, schizophrenia, animal models, 14-3-3 proteins

## Abstract

The N-methyl-d-aspartate receptor (NMDAR) hypofunction hypothesis has been proposed to help understand the etiology and pathophysiology of schizophrenia. This hypothesis was based on early observations that NMDAR antagonists could induce a full range of symptoms of schizophrenia in normal human subjects. Accumulating evidence in humans and animal studies points to NMDAR hypofunctionality as a convergence point for various symptoms of schizophrenia. Here we review animal models of NMDAR hypofunction generated by pharmacological and genetic approaches, and how they relate to the pathophysiology of schizophrenia. In addition, we discuss the limitations of animal models of NMDAR hypofunction and their potential utility for therapeutic applications.

## Introduction

Schizophrenia is a debilitating psychiatric disease that affects ~1% of the world population and places a major socio-economic burden (Blot et al., 2013). Patients exhibit positive and negative symptoms, as well as cognitive impairments, which typically emerge at early adolescence and worsen over time (Krystal et al., 1994; Morgan and Curran, 2006; Javitt, 2007; Howes et al., 2015). Both clinical and basic research suggest that schizophrenia is a neurodevelopmental disorder involving a variety of susceptibility genes, environmental risk factors, and epigenetic alterations (Cardno et al., 1999; Cannon et al., 2002; Shi et al., 2008; Walsh et al., 2008; Jaaro-Peled et al., 2009; Singh and O'Reilly, 2009), but it is unclear how these factors may contribute to the development of schizophrenic symptoms (Lewis and Gonzalez-Burgos, 2008; Faludi and Mirnics, 2011). Moreover, limited understanding on the pathophysiological mechanisms of schizophrenic brain has hindered the development of effective treatment for this disease. Current therapeutics is limited to antipsychotics (APDs) that mainly reduce positive symptoms but are ineffective for treating persistent negative or cognitive symptoms, which are often present before the onset of positive symptoms and lead to long-term functional impairments in schizophrenia patients (Howes et al., 2012; Citrome, 2014).

The glutamate hypothesis first emerged in the 1980s, and the N-methyl-D-aspartate receptor (NMDAR) hypofunction model was proposed afterwards upon observing that NMDAR antagonists could recapitulate a full range of positive, negative, and cognitive symptoms of schizophrenia in normal human subjects (Anis et al., 1983; Krystal et al., 1994). Consistent with this initial observation, findings from clinical, pharmacologic, and genetic studies suggested that NMDAR hypofunction may be one of the pathophysiological mechanism for schizophrenia (Goff and Coyle, 2001; Moghaddam, 2003; Jones et al., 2011; Javitt et al., 2012; Lin et al., 2012). NMDARs are ionotropic glutamate receptors that are comprised of the two obligatory NR1 subunits and two NR2 and/or NR3 subunits (Laurie and Seeburg, 1994). They function as a “coincidence detector” of pre- and post-synaptic activity and have crucial roles in glutamatergic neurotransmission, local rhythmic activity, and synaptic plasticity (Collingridge et al., 1988; Olney and Farber, 1995; Jensen and Lisman, 1996; Fellin et al., 2009). Thus, NMDARs are known to modulate cognition, memory, and higher-order brain functions (Moghaddam et al., 1997; Adams and Moghaddam, 1998; Palmer et al., 2008; Collingridge et al., 2013). In the last several decades, animal models of NMDAR hypofunction have been widely utilized to study the neurobiology of schizophrenia, as well as to test drugs for treating symptoms of schizophrenia. Here we review current literatures on animal models of NMDAR hypofunction using pharmacological and genetic approaches to induce NMDAR hypofunctionality.

## Pharmacological Approaches to Induce NMDAR Hypofunction

### Phencyclidine

Phencyclidine (PCP) is an abused drug that acts as a non-competitive blocker of NMDARs at lower doses. PCP administration was found to produce hallucinogenic activity in healthy human subjects (Itil et al., 1967). This had led to further investigation of the pathophysiological mechanisms for PCP-induced behavioral changes, particularly through inhibition of NMDARs (Carlsson and Carlsson, 1990; Johnson and Jones, 1990; Javitt and Zukin, 1991; Olney and Farber, 1995). To date, a wide range of animal studies have been conducted with different PCP administration regimen (acute, subchronic, or chronic), dosage, and co-administration with other drugs. The effects of PCP in animals at the molecular, electrophysiological, and behavioral levels were assessed by a variety of approaches.

In rodents, behaviors associated with positive symptoms of schizophrenia are assayed as novelty-induced hyperlocomotion in the open field test. Negative symptom-like behavioral alterations correlate with social withdrawal and anhedonia, which are commonly evaluated by social interaction, sucrose preference and forced swim tests (FST) (Sams-Dodd, 1996). Cognitive impairments are assessed using a variety of behavioral assays including radial arm maze, Morris water maze, Y-maze, T-maze, novel object recognition test, avoidance task learning test, attentional set-shifting task, modified hole board task, object and object-in-context recognition memory task, five-choice serial-reaction time test, and contextual fear conditioning. Moreover, the endophenotype corresponding to sensorimotor gating deficits in schizophrenics is detected as impaired prepulse inhibition (PPI) of acoustic startle response (McKibben et al., 2010). Acute administration of PCP induces a full spectrum of behavioral changes associated with positive, negative and cognitive symptoms of schizophrenia. Positive symptoms-related behaviors exhibited in acute PCP animal models include increased stereotypic behavior and ataxia, but there are conflicting reports regarding changes in locomotor activity. Negative symptoms-related behaviors are reflected as reduced social interaction, while cognitive deficits range from impaired latent learning, deficits in attention and cognitive flexibility to decreased sensorimotor gating (Sturgeon et al., 1979; Nabeshima et al., 1986; Mansbach and Geyer, 1989; Sams-Dodd, 1995, 1996; Martinez et al., 1999; Noda et al., 2001; Abdul-Monim et al., 2003; Egerton et al., 2005). These behavioral changes are accompanied by increased brain metabolism in areas of cortex, basal ganglia, and thalamus, decreased parvalbumin (PV) mRNA expression in the hippocampus and dorsal reticular nucleus of the thalamus, and altered zinc finger protein 225 (zif268) mRNA expression in the infralimbic cortex (Martinez et al., 1999; Egerton et al., 2005). Another study showed that acute PCP administration during early postnatal time period results in loss of PV-containing neurons from the primary somatosensory, motor, and retrosplenial cortices (Wang et al., 2008).

Subchronic (2–14 days) and chronic (15 days or longer) administration of PCP also generated a variety of schizophrenia-associated behavioral phenotypes (Noda et al., 1995; Sams-Dodd, 1995, 1996; Jentsch et al., 1997b, 1998; Stefani and Moghaddam, 2002; Balla et al., 2003; Abdul-Monim et al., 2006, 2007; Grayson et al., 2007; Egerton et al., 2008; Jenkins et al., 2008; Brigman et al., 2009; McKibben et al., 2010). Functional alterations induced by subchronic and chronic administration of PCP include reduced numbers of PV+ neurons in the hippocampus and frontal cortex. However, there are inconsistent reports on the PCP-induced changes in dopamine (DA) systems, as some studies observed potentiation of amphetamine-induced DA release and decreased basal DA metabolism in the prefrontal cortex (PFC) (Jentsch et al., 1997a; Balla et al., 2003; Cochran et al., 2003; Abdul-Monim et al., 2007; Jenkins et al., 2008; McKibben et al., 2010), whereas others reported reduced mesoprefrontal DA utilization (Jentsch et al., 1997a,b). In schizophrenia, dysregulation of DA systems is characterized by cortical hypodopaminergia and subcortical hyperdopaminergia (Slifstein et al., 2015). In that respect, there seems to be a discrepancy between the pharmacological rodent model and the disease.

Compared with acute exposure to PCP, subchronic and chronic exposure to PCP induces a more overt and sustained schizophrenia-like state in both rodents and in humans. As it provides a wider time frame to study long-lasting changes related to symptoms of schizophrenia, subchronic, and chronic PCP treated animal models are more commonly used to study the NMDAR hypofunctionality (Rainey and Crowder, 1975; Allen and Young, 1978; Cosgrove and Newell, 1991). In particular, these models have been applied to study the effects of NMDAR blockade on negative and cognitive symptoms-related behaviors, as well as to investigate the potential neurochemical and neuroanatomical basis underlying these behavioral changes (Jentsch and Roth, 1999; Lewis and Levitt, 2002; Table 1).

**Table 1:** Comparison of animal models of phencyclidine.

**Corresponding to SCZ**	**Dose/Regimen**	**Sex/Strain**	**Behavioral deficits**	**Structural and neurochemical changes**	**References**
Positive	Acute (2.5–15 mg/kg)	Male Sprague-Dawley Rats	Hyperlocomotor activity, increased stereotypy and ataxia	Not tested	Sturgeon et al., 1979
	Acute (0.9–29 μmol/kg) or (0.5–10 mg/kg)	Male Wistar Rats	Decreased locomotor activity, increased stereotypy and ataxia	Not tested	Sams-Dodd, 1995, 1996
	Subchronic (0.25–8 mg/kg for 5 days) or (0.9–29 μmol/kg for 3 days)	Male Wistar Rats	Hyperlocomotor activity, increased stereotypy and ataxia	Stereotyped behavior reversed by chronic clozapine administration	Sams-Dodd, 1995, 1996
	Subchronic (5 mg/kg bi-daily for 7 days)	Male Sprague-Dawley Rats	Hyperlocomotor activity, increased sensitivity to amphetamine	Reduced prefrontal cortical DA utilization, prolonged hypoactivity of mesocortical DA neurons and hyper-responsivity of mesolimbic DA neurons	Jentsch et al., 1998
	Subchronic (5 mg/kg for 3–14 days)	Male Sprague-Dawley Rats	Hyperlocomotor activity	Dose-dependent enhancement in amphetamine-induced DA release in the PFC	Balla et al., 2003
Negative	Acute (0.45–57 μmol/kg)	Male Wistar Rats	Decreased social behavior	Not tested	Sams-Dodd, 1995, 1996
	Subchronic (0.25–8.0 mg/kg for 5 days) or (0.9–29 μmol/kg for 3 days)	Male Wistar Rats	Concomitant reductions in the explorative and social behaviors	Social isolation reversed by chronic clozapine administration	Sams-Dodd, 1995, 1996
	Subchronic (10 mg/kg for 14 days)	Male ddY mice	Increased time immobile in the FST	FST effect was reversed by atypical APDs, risperidone and clozapine	Noda et al., 1995
	Subchronic (2 mg/kg bi-daily for 7 days)	Male hooded-Listar Rats	Disturbances in social interaction	Reduced PV+ neurons in the hippocampus with significant reductions localized to the CA1 and DG regions of the hippocampus	Jenkins et al., 2008
	Subchronic (5 mg/kg bi-daily for 7 days)	Male C57/BL6 WT mice	Partial deficits in social behavior	Not tested	Brigman et al., 2009
Cognition	Acute (0.25, 0.75, 1.5, 10 mg/kg)	Male and female Sprague Dawley Rats	Decreased sensorimotor gating	PCP increases brain metabolism in areas of cortex, basal ganglia, and thalamus; enhances norepinephrine release and inhibits striatal synaptosomal DA	Martinez et al., 1999
	Acute (0.5 mg/kg, 1 mg/kg)	Male ddY mice	Impaired latent learning in a one-trial water-finding task	Sigma1 receptor ligands attenuated the PCP-induced latent learning impairment	Noda et al., 2001
	Acute (1.0, 1.5 mg/kg)	Female Hooded Listar Rats (socially isolated from P21)	Impaired reversal task performance, increased locomotor activity from isolated rats compared to socially reared rats	Atypical APD (ziprasidone) reversed impairments caused by PCP	Abdul-Monim et al., 2003
	Acute (2.58 mg/kg)	Male hooded Long-Evans Rats	Deficits in attentional set-shifting	Altered zif268 mRNA expression in the infralimbic cortex and PV mRNA expression in the dorsal reticular nucleus of the thalamus	Egerton et al., 2005
	Acute (10 mg/kg on P7)	Male Sprague Dawley Rats	Not tested	Loss of PV containing neurons from primary somatosensory, motor, and retrosplenial cortices	Wang et al., 2008
	Subchronic (10 mg/kg for 10 days, 5 mg/kg bi-daily for 7 days)	Male Sprague-Dawley Rats	Impaired spatial delayed alternation task	Decreased basal DA utilization in the PFC	Jentsch et al., 1997b
	Subchronic (2 mg/kg bi-daily for 7 days)	Female hooded-Listar Rats	Enduring, persistent deficits in reversal learning	Cognitive deficits are attenuated by treating with atypical but not classical APDs	Abdul-Monim et al., 2006
	Subchronic (2 mg/kg bi-daily for 7 days)	Female hooded-Listar Rats	Deficits in reversal learning	Reduced PV+ neurons in the hippocampus, frontal cortex with reduced in the motor area 1 (M1) and increases in motor area 2 (M2) region and cingulate cortex	Abdul-Monim et al., 2007
	Subchronic (2 mg/kg bi-daily for 7 days)	Female hooded-Listar Rats	Deficits in novel object recognition test	Clozapine and risperidone attenuated PCP-induced impairments	Grayson et al., 2007
	Subchronic (2.6 mg/kg for 5 days)	Male hooded Long-Evans Rats	Deficits in attentional set-shifting and sensorimotor gating	Transient metabolic alterations present across multiple brain regions	Egerton et al., 2008
	Subchronic (2 mg/kg bi-daily for 7 days)	Male hooded-Listar Rats	Deficits in novel object recognition test	Reduction in PV+ neurons in the PFC with specific deficits observed in the prelimbic region	McKibben et al., 2010
	Chronic intermittent exposure (5 mg/kg bi-daily for 5 days, 3 days after 10 days)	Male Sprague-Dawley Rats	No long-term impairment in T-maze alternation performance	Not tested	Stefani and Moghaddam, 2002
	Chronic intermittent exposure(0.86 or 2.58 mg/kg for 5 days and post-8–26 days)	Male hooded Long-Evans Rats	Not tested	Metabolic hypofunction in PFC increase glutamate release; initial increase in glucose utilization or uptake, activating non-NMDARs	Cochran et al., 2003
	Chronic intermittent exposure(2.6 mg/kg for 5 days and post-8–26 days)	Male hooded Long-Evans Rats	Deficits in attentional set-shifting	Transient metabolic alterations present across multiple brain regions	Egerton et al., 2008

### Ketamine

Ketamine was first synthesized as a PCP derivative back in the 1960s in an effort to minimize the side effects and neurotoxicity of PCP (Maddox et al., 1965). As a noncompetitive NMDAR antagonist, ketamine acts by specifically binding to a site near the channel pore (Roth et al., 2013). Ketamine was found to be safe and effective even with repeated administration with minimal side effects and was soon used as a clinical anesthetic (Domino et al., 1965; Corssen and Domino, 1966; Li and Vlisides, 2016). Ketamine administration has often been used in animal studies as a preclinical model to test the effects of APDs and novel compounds since atypical APDs are found to be effective in blocking ketamine's behavioral effects in both humans and rodents (Jentsch and Roth, 1999; Krystal et al., 1999, 2005; Becker et al., 2003; Lees et al., 2004; Gilmour et al., 2009; Neill et al., 2010). However, apart from the blockade of NMDARs, ketamine can act on a wide-range of targets in various cellular processes (Li and Vlisides, 2016).

Acute ketamine administration in animals results in cognitive deficits including reduced sensorimotor gating, spatial learning and memory impairments, along with changes in theta and gamma band activity (Verma and Moghaddam, 1996; de Bruin et al., 1999; Ehrlichman et al., 2009; Kittelberger et al., 2012; Szlachta et al., 2017; Coronel-Oliveros and Pacheco-Calderon, 2018). In contrast to cortical hypodopaminergic state observed in schizophrenia, these cognitive deficits are accompanied by increased DA release in the PFC (Verma and Moghaddam, 1996). Other studies also reported that cognitive deficits are associated with increased frontal cortical blood flow (Ingvar and Franzen, 1974; Vollenweider et al., 1997). Moreover, acute ketamine administration induces hyperlocomotor activity and stereotypic behaviors, which might be attributed to increased DA and serotonin turnover in the striatum and cortex (Irifune et al., 1991; Chatterjee et al., 2011, 2012; Coronel-Oliveros and Pacheco-Calderon, 2018). However, there are inconsistent reports as to the effect of acute ketamine administration on negative symptoms-related behaviors based on social interaction tests (Silvestre et al., 1997; Chatterjee et al., 2011; Coronel-Oliveros and Pacheco-Calderon, 2018). In fact, ketamine is known to have anti-depressant effects at low doses, which may oppose the induction of negative symptoms-related behaviors in animals (Zanos and Gould, 2018).

Subchronic ketamine administration, however, can induce negative symptoms-related behaviors, in addition to positive symptoms-related behaviors. These include increased time spent immobile during the forced swim test (FST), hyperlocomotor activity, and stereotypy (Becker et al., 2003; Chatterjee et al., 2011, 2012). Hyperlocomotor activity exhibited after a subchronic dose of ketamine is attributed to increased DA and serotonin levels in the cortex and striatum, which is thought to result from alternations in gene expression of DA and serotonin receptors (Chatterjee et al., 2011, 2012). On the other hand, negative symptom-related behavior in both acute and subchronic ketamine administration is associated with decreased levels of glycine in the striatum, hippocampus, and cortex (Chatterjee et al., 2012). This is consistent with previous findings from schizophrenia patients that linked reduced levels of glycine to negative symptoms of schizophrenia and increased levels of glycine for the treatment of negative symptoms (Javitt, 2010).

Subchronic ketamine administration also induce cognitive deficits, such as reversal learning and long-term spatial memory impairments (Featherstone et al., 2012; Szlachta et al., 2017). In addition, chronic ketamine administration results in decreased PV interneuron density in the hippocampus (Keilhoff et al., 2004; Kittelberger et al., 2012). Fast-spiking PV interneurons are important for encoding and storage of information required for working memory, and dysfunction in fast-spiking PV interneurons are known to cause cognitive deficits, potentially through disrupting theta and gamma oscillations (Bartos et al., 2007). Indeed, subchronic ketamine administration induce cognitive deficits that are accompanied by alterations in theta and gamma oscillation from the hippocampus and PFC (Featherstone et al., 2012). Moreover, audioradiographic imaging studies have reported dysconnectivity between PFC and hippocampus in both acute and subchronic ketamine animal models (Dawson et al., 2013).

Overall, ketamine can induce behavioral alterations associated with schizophrenia symptoms but with less excitotoxicity than PCP. When comparing treatment regimens, subchronic ketamine treatment models are more widely used, since it can induce persistent negative and cognitive symptoms-related behaviors with hallmark features of dysfunction in neuronal circuitry (Lahti et al., 2001; Morgan et al., 2009). However, behavioral deficits and cortical dysfunctions cannot be attributed to changes only in the glutamatergic system as it may involve changes in other neurotransmitter systems such as DA and serotonin (Grace, 2012; Table 2).

**Table 2:** Comparison of animal models of ketamine.

**Corresponding to SCZ**	**Dose/Regimen**	**Sex/Strain**	**Behavioral deficits**	**Structural and neurochemical changes**	**References**
Positive	Acute (3–150 mg/kg)	Male ddY mice	Hyperlocomotor activity	Low dose ketamine increased DA turnover in the nucleus accumbens, high dose increased DA, norepinephrine, and serotonin turnover in many brain regions; hyperlocomotor activity attenuated by haloperidol and destruction of catecholaminergic terminals via 6-hydroxydopamine	Irifune et al., 1991
	Acute (100 mg/kg)	Male Swiss Webster Rats	Hyperlocomotor activity and stereotypy	Not tested	Chatterjee et al., 2011
	Acute (100 mg/kg)	Male Swiss Webster Rats	Not tested	Increased DA turnover and serotonin turnover in the striatum and cortex, increased glutamate levels in cortex and decreased glutamine levels in the striatum, decreased glycine levels in the cortex, striatum, and hippocampus	Chatterjee et al., 2012
	Acute (60 mg/kg and 20 mg/kg every 20 min for 3 h) administered on E14	Male Sprague-Dawley Rats	Hyperlocomotor activity in juvenile ketamine group as a response to stress/novelty, increased stereotypic behavior and agitation	Adult dorsolateral hippocampus shows a reduction of the CA3 region thickness	Coronel-Oliveros and Pacheco-Calderon, 2018
	Subchronic (30 mg/kg for 5 days)	Male Sprague-Dawley Rats	Enhancement in locomotor activity	Increased D2R binding in the hippocampus and decreased glutamate receptor binding in the frontal cortex. DA transporter (DAT) density increased in the striatum and serotonin transporter density increased in the striatum, hippocampus, and the frontal cortex	Becker et al., 2003
	Subhronic (100 mg/kg for 10 days)	Male Swiss Webster Rats	Hyperlocomotor activity	Not tested	Chatterjee et al., 2011
	Subchronic (100 mg/kg for 10 days)	Male Swiss Webster Rats	Not tested	Increased DA turnover and serotonin turnover in the striatum and cortex; increased gene expression of D1R, D2R, DAT, tyrosine hydroxylase, 5HT1A and 5HT2A receptors with decreased gene expression of 5HT2C receptors in the cortex	Chatterjee et al., 2012
Negative	Acute (7 mg/kg)	Male Wistar Rats	Decreased social interaction, anxiogenic-like effect	Not tested	Silvestre et al., 1997
	Acute (100 mg/kg)	Male Swiss Webster Rats	Reduced “transfer latency” time in passive avoidance test with no effect in the FST	Not tested	Chatterjee et al., 2011
	Acute (60 mg/kg and 20 mg/kg every 20 min for 3 h) administered on E14	Male Sprague-Dawley Rats	Social withdrawal, depression, and anxiety-like behaviors	Adult dorsolateral hippocampus shows a reduction of the CA3 region thickness	Coronel-Oliveros and Pacheco-Calderon, 2018
	Subchronic (30 mg/kg for 5 days)	Male Sprague-Dawley Rats	Not tested	Increased D2R binding in the hippocampus and decreased glutamate receptor binding in the frontal cortex. DAT density increased in the striatum and serotonin transporter density increased in the striatum, hippocampus, and the frontal cortex	Becker et al., 2003
	Subchronic (100 mg/kg for 10 days)	Male Swiss Webster Rats	Enhanced immobility during the FST paradigm	Pre-treatment with clozapine and risperidone attenuated enhanced immobility period in the FST	Chatterjee et al., 2011
	Subchronic (100 mg/kg for 10 days)	Male Swiss Webster Rats	Not tested	Increased DA turnover and serotonin turnover in the striatum and cortex; increased gene expression of D1R, D2R, DAT, tyrosine hydroxylase, 5HT1A and 5HT2A receptors with decreased gene expression of 5HT2C receptors in the cortex, increased glutamate levels in cortex and decreased glutamine levels in the striatum, decreased glycine levels in the cortex, striatum, and hippocampus	Chatterjee et al., 2012
Cognition	Acute (10–30 mg/kg)	Male Sprague-Dawley Rats	Impairment in spatial delayed alternation task	Increased release of DA in the PFC as compared to striatum; haloperidol and raclopride was able to reverse the ketamine-induced effect	Verma and Moghaddam, 1996
	Acute (2.5 mg/kg, 10 mg/kg)	Male hooded-Listar Rats	Disrupted PPI of the startle response to the extent that prepulse facilitation occurred	Not tested	de Bruin et al., 1999
	Acute (20 mg/kg)	Male C57/BL6 WT mice	Increased basal power in gamma band and decreased evoked power in the theta band	The increase in basal gamma was not blocked by treatment with conventional APDs	Ehrlichman et al., 2009
	Acute (10 mg/kg)	Male Sprague-Dawley Rats	Increased gamma power in both CA1 and DG in the hippocampus	Theta peak shifted to higher frequency, whereas 5–10 Hz theta power decreased in the CA1 and remained high in the DG	Kittelberger et al., 2012
	Acute (0.3, 3 mg/kg)	Male Sprague-Dawley Rats	Impairment in attentional set shifting task	Clozapine reversed the ketamine-induced effect	Szlachta et al., 2017
	Acute (60 mg/kg and 20 mg/kg every 20 min for 3 h) administered on E14	Male Sprague-Dawley Rats	Spatial memory impairments in adulthood accompanied by anxiety-like behavior	Adult dorsolateral hippocampus shows a reduction of the CA3 region thickness	Coronel-Oliveros and Pacheco-Calderon, 2018
	Subchronic (30 mg/kg for 5 days)	Male Sprague-Dawley Rats	Not tested	Increased density of reduced nicotinamide adenine dinucleotide phosphate diaphorase (NADPHd), neuronal nitric oxide synthase (nNOS), and cFOS+ hippocampal interneurons; decreased PV+ neurons	Keilhoff et al., 2004
	Subchronic (30 mg/kg for 2 days)	Male C57/BL6 mice	Not tested	Loss of PV and GAD67 expression in the PFC. Increase in brain superoxide due to activation in neurons of reduced nicotinamide adenine dinucleotide phosphate (NADPH) oxidase	Behrens et al., 2007
	Subchronic (20 mg/kg for 14 days)	Male 3H/HeHsD mice	Disruption in reversal learning and spatial memory, reduction in stimulus-evoked theta oscillations, stable alterations in EEG/ERP responses	Decreased expression of glial-specific glutamate transporter (GLT-1). Increased astrocyte proliferation and decreased expression of excitatory amino acid transporter 2 (EAAT2) in the PFC	Featherstone et al., 2012
	Subchronic (30 mg/kg for 5 days)	Male Sprague-Dawley Rats	Steady decline in theta and gamma oscillations over 2–4 weeks after treatment in both CA1 and DG	Decreased numbers of PV interneurons in the hippocampus	Kittelberger et al., 2012
	Subchronic (0.3 mg/kg for 7 days)	Male Sprague-Dawley Rats	Impairment in attentional set shifting task	Clozapine reversed the effect of ketamine after chronic treatment	Szlachta et al., 2017

### MK-801

Also known as dizocilpine, MK-801 is a non-competitive NMDAR antagonist that blocks the channel in a use- and voltage-dependent manner (Foster and Fagg, 1987; Huettner and Bean, 1988). Like ketamine, MK-801 seems to preferentially act on GABAergic interneurons. This selective action of MK-801 on interneurons reduces inhibitory influence on excitatory pyramidal neurons, leading to hyperexcitation in the PFC neuronal circuit (Yonezawa et al., 1998; Homayoun and Moghaddam, 2007). However, more recent studies showed that local MK-801 infusion into the PFC does not directly produce disinhibition (Gratton et al., 1987; Suzuki et al., 2002; Jodo et al., 2005), suggesting that hyper-glutamatergia in the PFC may require MK-801's effects in other brain regions such as the hippocampus (Jodo, 2013; Nakazawa et al., 2017). Moreover, MK-801 has substantially longer action of NMDAR blockade in rodents and higher specificity for NMDARs than ketamine (Miyamoto et al., 2000; Pinault, 2008; Hakami et al., 2009).

Perhaps due to its prolonged action, high potency and specificity for NMDARs, MK-801 can produce a full range of schizophrenia-related behavioral phenotypes in both acute and chronic treatment models. In rodents, acute MK-801 administration induces hyperlocomotor activity, decreased social interaction, and impairments in cognitive flexibility, latent learning, long-term spatial memory, working memory, and sensorimotor gating (Yamada et al., 1996; Bast et al., 2000; Noda et al., 2001; Harris et al., 2003; Rung et al., 2005; Abekawa et al., 2007; Manahan-Vaughan et al., 2008; Zou et al., 2008; Wiescholleck and Manahan-Vaughan, 2012). Cognitive deficits induced by acute MK-801 administration are associated with impaired long term potentiation (LTP) (Manahan-Vaughan et al., 2008; Wiescholleck and Manahan-Vaughan, 2012). Acute MK-801 treatment also results in decreased PV mRNA expression in the PFC, hippocampus, bed nucleus of the stria terminalis (BNST) amygdala, orbitofrontal and entorhinal cortex, with no changes in glutamate decarboxylase 67 kDa (GAD67) mRNA expression (Abekawa et al., 2007; Romon et al., 2011). Acute MK-801 administration is accompanied by increased gamma power in the hippocampus and decreased beta band power in the hippocampus (Kittelberger et al., 2012; Sullivan et al., 2015). In addition, acute ketamine treatment leads to increased synapse-associated protein 90/postsynaptic density protein 95 (SAP90/PSD95) and protein kinase C gamma (PKCγ) mRNA expression from cortical regions, decreased NR2C protein expression from the entorhinal cortex, and decreased NR2B protein expression from the parietal cortex (Linden et al., 2001).

Chronic MK-801 treatment also results in dysfunctional GABAergic interneurons as indicated by reduced PV interneuron density in the hippocampus (Braun et al., 2007). Similarly to acute treatment, chronic and subchronic MK-801 treatment leads to cognitive deficits (Latysheva and Rayevsky, 2003; Stefani and Moghaddam, 2005; Rujescu et al., 2006; Li et al., 2011; Xiu et al., 2014; Liu et al., 2017; Unal et al., 2018). In addition, chronic MK-801 treatment leads to sensorimotor gating and social interaction deficits, although there are controversial results on locomotor activity (Latysheva and Rayevsky, 2003; Eyjolfsson et al., 2006; Xiu et al., 2014; Unal et al., 2018). Another study showed that chronic MK-801 induce increased anxiety-like behavior and working memory deficits, which was accompanied by degenerative changes of myelin sheaths, decreased white matter and corpus callosum volume (Xiu et al., 2014). Early postnatal chronic MK-801 treatment model reported reduced locomotor activity, decreased rearing behavior, exploratory behavior, increased anxiety-like behavior as well as learning impairments (Latysheva and Rayevsky, 2003; Table 3). Results from MK-801 administration in awake rats suggest that NMDAR inhibition causes cortical excitation by disinhibition of pyramidal neurons in the PFC (Homayoun and Moghaddam, 2007). In addition, studies consistently report that chronic MK-801 administration leads to increased dopaminergic and serotonergic activity in the frontal cortex, nucleus accumbens and striatum (Loscher et al., 1991). As discussed earlier, an increased dopaminergic activity in the frontal cortex by chronic MK-801 administration may not be consistent with the cortical hypodopaminergic state in schizophrenia patients, which further points to the limitations in the use of pharmacological rodent models to recapitulate the dysregulation of DA systems.

**Table 3:** Comparison of animal models of MK-801.

**Corresponding to SCZ**	**Dose/Regimen**	**Sex/Strain**	**Behavioral deficits**	**Structural and neurochemical changes**	**References**
Positive	Acute (0.5 mg/kg bi-daily at P7)	Male and Female Sprague-Dawley Rats	Females show hyperlocomotor activity	Reduced volume and neuronal numbers in the hippocampus, altered hippocampal NR1 protein expression, decreased synaptophysin mRNA expression in the thalamus	Harris et al., 2003
	Acute (0.1, 0.2, 0.3 mg/kg)	Male Sprague-Dawley Rats	Hyperlocomotor activity at higher doses	Not tested	Rung et al., 2005
	Acute prenatal exposure (0.2 mg/kg at E15–E18)	Male and female Sprague-Dawley Rats	Enhanced PCP-induced hyperlocomotion on P63 but not on P35	Reduction in PV+ neurons in the mPFC at both P35 and P63	Abekawa et al., 2007
	Acute (5 mg/kg)	Male Wistar Rats	Hyperlocomotor activity, increased stereotypy, and ataxia	LTP impaired in rats 7 days after injection accompanied by inability to form spatial memory; LTP was recovered 4 weeks after treatment	Manahan-Vaughan et al., 2008
	Subchronic (0.1 mg/kg for 6 days)	Male Sprague-Dawley Rats	Slightly increased locomotor activity	Glutamate and glutamine in the temporal lobe increased in neuronal and astrocytic metabolism	Eyjolfsson et al., 2006
	Subchronic (1 mg/kg for 14 days)	Male C57/Bl6 mice	Hyperlocomotor activity and increased anxiety-like behavior	Degenerative changes of myelin sheaths, total white matter volume and corpus callosum volume decreased	Xiu et al., 2014
	Chronic postnatal (0.05 mg/kg from P7–P49)	Male Wistar Rats	Hyperlocomotor activity, decreased rearing and exploratory behavior, increased anxiety-like behavior	Not tested	Latysheva and Rayevsky, 2003
Negative	Acute (0.1 mg/kg)	Male ICR mice	Decreased social interaction, Females show hyperlocomotor activity	Pretreatment with MK801 resulted in both a reduction of social investigation and discriminative capacities	Zou et al., 2008
	Subchronic (0.2 mg/kg for 14 days)	Male Wistar Hannover rats	Social deficits seen through decreased exploratory behavior and increased avoidance behavior	Agmatine partially reversed social deficits while risperidone completely reversed social deficits	Unal et al., 2018
Cognition	Acute (0.1 mg/kg)	Male ddY mice	Impaired spatial working memory and long-term memory	Decreased nitric oxide (NO) synthase activity in the cerebral cortex/hippocampus, pretreatment with dibutyryl cyclic GMP and L-arginine ameliorated the MK-801-induced spatial working memory deficit	Yamada et al., 1996
	Acute (0.05, 0.075, 0.1 mg/kg)	Male Sprague-Dawley and Male Wistar Rats	Decreased sensorimotor gating in both rat strains, bilateral micro infusion of MK-801 into the ventral hippocampus did not affect PPI	Neither clozapine nor haloperidol antagonized MK-801-induced PPI in either rat strain	Bast et al., 2000
	Acute (5 mg/kg)	Male Wistar Rats	Not tested	Increased mRNA levels of SAP90/PSD95 and gamma isoform of PKC gamma in cortical regions, increased synapse-associated protein-97 (SAP97) mRNA levels in the entorhinal cortex layer III, decreased expression of NR2C in the entorhinal and NR2B in the parietal cortex	Linden et al., 2001
	Acute (0.05 mg/kg)	Male ddY mice	Impairment in latent learning in a one-trial water-finding task	Sigma1 receptor agonist (SA4503) attenuated the MK-801-induced impairment of latent learning	Noda et al., 2001
	Acute (0.5 mg/kg bi-daily at P7)	Male and Female Sprague-Dawley Rats	Females show PPI deficits	Reduced volume and neuronal numbers in the hippocampus, altered hippocampal NR1 protein expression, decreased synaptophysin mRNA expression in the thalamus	Harris et al., 2003
	Acute (6 mg/kg)	Male C57/BL6 WT mice	Not tested	Kainite-induced gamma frequency field oscillations in animals increased in power and depolarized resting membrane potentials were higher in CA1 pyramidal cells	Kehrer et al., 2007
	Acute (5 mg/kg)	Male Wistar Rats	Disruption in PPI and deficits in long-term spatial memory	LTP impaired in rats 7 days after injection accompanied by inability to form spatial memory; LTP was recovered 4 weeks after treatment	Manahan-Vaughan et al., 2008
	Acute (1 mg/kg)	Male Wistar Rats	Not tested	Decreased PV mRNA expression from mPFC, orbitofrontal and entorhinal cortices, hippocampus, and BNST of amygdala with no change in GAD67 in any brain regions	Romon et al., 2011
	Acute (0.2 mg/kg)	Male Sprague-Dawley Rats	Increased gamma power in both CA1 and DG of the hippocampus	Theta peak shifted to higher frequency, whereas 5–10 Hz theta power decreased in CA1 and remained high in DG	Kittelberger et al., 2012
	Acute (5 mg/kg)	Male Wistar Rats	Impaired object recognition memory; restored by phosphodiesterase type 4 (PDE4) inhibitor, rolipram	Impairment of LTP in the DG, which was restored by rolipram	Wiescholleck and Manahan-Vaughan, 2012
	Acute (0.1 mg/kg)	Male Sprague-Dawley Rats	Increased gamma power and decreased beta band power in the hippocampus	Loss of GABAA activity	Sullivan et al., 2015
	Subchronic postnatal (0.1 mg/kg from P7–P11)	Male Sprague Dawley Rats	Impaired cognitive flexibility and working memory	Not tested	Stefani and Moghaddam, 2005
	Subchronic (0.02 mg/kg for 14 days from P35–49)	Male Long Evans Rats	Working memory deficits in a modified Hole Board task	Altered the expression of NR1 splice variants, decreased expression of NR2B and NR2C in the hippocampus, decreased numbers of PV+ neurons in the hippocampus, altered recurrent inhibition of pyramidal cells	Rujescu et al., 2006
	Subchronic (0.05, 0.1, 0.2 mg/kg × 14 days) at P28	Male Sprague Dawley Rats	Impairments in spatial working memory and associative memory	Not tested	Li et al., 2011
	Subchronic (1 mg/kg for 14 days)	Male C57/BL6 mice	Spatial working memory deficits	Degenerative changes of myelin sheaths, total white matter volume, and corpus callosum volume decreased	Xiu et al., 2014
	Subchronic (0.2 mg/kg for 14 days)	Male Wistar Hannover rats	Decreased PPI and visual recognition memory deficit	Agmatine and risperidone both reversed visual recognition memory deficit	Unal et al., 2018
	Chronic postnatal (0.05 mg/kg from P7–P49)	Male Wistar Rats	Slower spatial learning	Not tested	Latysheva and Rayevsky, 2003
	Chronic (0.02 mg/kg for 21 days)	Male Long-Evans Rats	Not tested	Reduction in PV+ neurons in the hippocampus; no change in calretinin+ neurons nor NADPH staining	Braun et al., 2007
	Chronic (0.1 mg/kg for 21 days)	Male Sprague Dawley Rats	Object-in-context recognition memory and reversal learning in the Morris water maze, reversed partially by olanzapine	Cognitive deficits rescued by olanzapine. Reduction in levels of NR1 and phosphorylated NR2B, GluA1, and PSD95 in the mPFC, restored levels of NR1 and phosphorylated NR2B by olanzapine	Liu et al., 2017

## Genetic Approaches to Induce NMDAR Hypofunction

### Knockout or Knockdown of NR1 Subunits

Based on genetic analyses, polymorphisms exist in both coding and promoter regions of glutamate receptor ionotropic NMDA type subunits (GRIN), impacting both NMDAR transcript levels and/or functions. Single nucleotide or dinucleotide-repeated polymorphisms of the NMDAR subunit genes, such as NR1 (GRIN1), NR2A (GRIN2A), and NR2B (GRIN2B), increase susceptibility to schizophrenia (Ohtsuki et al., 2001; Rice et al., 2001; Miyatake et al., 2002; Itokawa et al., 2003; Iwayama-Shigeno et al., 2005; Qin et al., 2005; Martucci et al., 2006; Tang et al., 2006; Zhao et al., 2006). Moreover, postmortem and genetic studies from schizophrenia patients support that abnormalities in glycine modulatory sites on the NMDARs may contribute to the pathophysiology of schizophrenia (Coyle, 2004).

Amongst various NMDAR subunits, NR1 is essential for formation and synaptic expression of NMDARs (Laurie and Seeburg, 1994). A complete loss of NR1 subunit in mice [NR1 knockout (KO)] is neonatally lethal, suggesting that NMDARs play a crucial role in early development (Forrest et al., 1994). To overcome neonatal lethality, a line of NR1 knockdown (KD) mice was generated by ectopic transgenic expression of one of the NR1 splice variants (NR1-1a) in the NR1 KO mice. This line of NR1 KD mice exhibits altered dendritic differentiation, branching, and somatosensory pattern, accompanied by increased axonal arborizations with faster and prematurely developed projection neurons of the corpus callosum (Iwasato et al., 1997; Lee et al., 2005). However, the average lifespan of this NR1 KD mice was dependent on the level of transgene expression. This limits the usage of these mice for further behavioral, molecular, and structural studies in adult mice. Another NR1 KD mice were created by inserting a neomycin cassette into an intronic region of the GRIN1 gene, leading to significantly lower levels of NR1 expression (Mohn et al., 1999). These mice are viable and exhibit a full range of behavioral phenotypes associated with schizophrenia, such as hyperlocomotor activity, stereotypy, self-injury, decreased anxiety-related behavior, reduced nest building, impaired social and sexual interactions, abnormal evoked response potentials (ERPs), cognitive inflexibility, abnormal selective attention, with spatial cognitive and sensorimotor gating deficits (Mohn et al., 1999; Duncan et al., 2004; Moy et al., 2006; Bickel et al., 2007; Dzirasa et al., 2009; Halene et al., 2009). These NR1 KD mice also exhibit reduced synapse-number in an age-dependent manner, decreased NMDA currents, reduced 2-deoxyglucose (DG) uptake in areas of the neocortex, increased amplitudes of auditory and visual ERPs, attenuated cortical and hippocampal theta-gamma phase coupling, increased dendritic length, and synapse-specific reductions in 14-3-3ε and disrupted in schizophrenia 1 (DISC1) protein expression (Duncan et al., 2002; Dzirasa et al., 2009; Halene et al., 2009; Ramsey et al., 2011). Others have also developed hypomorphic NR1 subunit mice with single or double point mutations in NMDAR glycine binding sites, GRIN1^D481^ and GRIN1^K483^. Hypomorphic NR1 mice with single point mutation in D481 (GRIN1^D481N^) are viable and exhibit increased startle reactivity, deficits in spatial recognition, spatial reference learning and memory, reduced sociability, anxiety, and sensitivity to NMDA-induced seizures, with impaired hippocampal LTP (Kew et al., 2000; Labrie et al., 2008). In contrast, GRIN1^K483Q^ mice are perinatally lethal (Kew et al., 2000). Subsequently, Ballard et al. developed the compound heterozygote mice with point mutations in both glycine binding sites. These mice (GRIN1^D481N/K483Q^) are viable and display hyperlocomotor activity, stereotypy, disrupted nesting behavior, spatial learning, and sensorimotor gating deficits. These behavioral changes are accompanied by striatal dopaminergic and serotonergic hyperfunction. Interestingly, deficits in hippocampal LTP are rescued by D-serine administration (Ballard et al., 2002).

To understand how NMDAR hypofunction affects behaviors at cellular and circuit levels, various animal models with brain region- and/or cell-type specific deletion of NR1 have been created. Forebrain excitatory neuron-specific NR1 KO mice exhibit impairments in spatial memory, encoding and flexible expression of non-spatial memory with slower acquisition of trace fear conditioning. These mice also exhibit degraded co-activation of CA1 place cells during exploration and lack NMDAR-mediated synaptic currents and LTP in CA1 synapses (McHugh et al., 1996; Tsien et al., 1996; Huerta et al., 2000; Rondi-Reig et al., 2001). Dentate-gyrus (DG) granule cells-specific NR1 KO mice display deficits in the process of pattern separation, which is accompanied by reduced firing rate from cornu ammonis 3 (CA3) pyramidal cells during context-specific modulation. These mice also exhibit deficits in spatial working memory accompanied by impaired LTP in both medial and lateral perforant path inputs to the DG (McHugh et al., 2007; Niewoehner et al., 2007). CA1-specific NR1 KD rats were created by injecting NR1 antisense RNA into the CA1 region of the hippocampus. These rats exhibit impairments in avoidance task learning and memory formation (Cheli et al., 2002, 2006). Similarly, CA3 pyramidal cells-specific NR1 KO mice exhibit deficits in associative memory recall accompanied by impaired NMDAR-dependent LTP at the commissural/associational (C/A) pathway (Nakazawa et al., 2002). Another CA3-specific NR1 KO mice, created by an adeno-associated virus (AAV)-induced deletion of the NR1 gene, exhibit increased impulsive behavior, learning impairments, and decreased social approach behavior (Rajji et al., 2006; Finlay et al., 2015).

Mice with deletion of NR1 from cortical and hippocampal GABAergic interneurons during early postnatal development exhibit hyperlocomotor activity, anhedonia-like and anxiety-like behaviors, mating and nest-building deficits, as well as social memory, spatial working memory, and prepulse inhibition deficits. These changes are accompanied by increased firing of cortical excitatory neurons with reduced neuronal synchrony. Overall, these findings support that NMDAR hypofunction in corticolimbic GABAergic interneurons during early postnatal development can lead to the development of schizophrenia-related behavioral phenotypes (Belforte et al., 2010). Furthermore, PV-specific NR1 KO mice were generated by targeted deletion of NR1 subunits from a subset of GABAergic interneurons. These mice exhibit deficits in spatial working, short- and long-term recognition memory, which are correlated with profound changes in neural activity related to cognition including increased gamma oscillation power and decreased theta oscillation power of local field potentials (LFP) in the hippocampus (Korotkova et al., 2010). Moreover, other groups reported that the PV-specific NR1 KO mice display blunted MK-801-induced hyperlocomotor activity, suggesting that NMDARs in PV interneurons may be the site of MK-801 action (Belforte et al., 2010; Carlen et al., 2012). However, a more recent study reported that these mice are not protected against behavioral effects of MK-801, as MK-801 administration leads to increased stereotypy and pronounced catalepsy, which confound the locomotor readout (Bygrave et al., 2016).

NR1 deletion in forebrain pyramidal neurons also results in behavioral changes associated with schizophrenia. The forebrain pyramidal cells-specific NR1 KO mice display hyperlocomotor activity and decreased self-care, as well as social and cognitive impairments (Tatard-Leitman et al., 2015). In addition, these mice exhibit decreased expression of dopamine D2 receptor (D2R) and G-protein-regulated inward-rectifier potassium channel 2 (GIRK2) in the forebrain, increased baseline gamma power and pyramidal cell excitability (Tatard-Leitman et al., 2015). However, mPFC and sensory cortex pyramidal cells-specific NR1 KO mice only exhibit PPI and short-term memory impairments, suggesting that deletion of NR1 subunit in pyramidal neurons of broader forebrain regions may be required to induce a full range of symptoms of schizophrenia (Kehrer et al., 2008). Taken together, these studies demonstrate that NR1-mediated deficits in either pyramidal or GABAergic neurons could cause an imbalance of excitation and inhibition in the cortical neural circuit, leading to development of behavioral phenotypes related to schizophrenia (Table 4).

**Table 4:** Genetic animal models of NMDAR hypofunctionality.

**Animal model**	**Positive symptom-like behavior**	**Negative symptom-like behavior**	**Sensorimotor gating deficits**	**Cognitive deficits**	**Structural and neurochemical changes**	**References**
NR1 subunit knockout	• Hyperlocomotor activity, stereotypy, and decreased anxiety-like behavior in NR1 KD mice • Hyperlocomotor activity and stereotypy in GRIN1 compound het mice • Hyperlocomotor activity in cortical and hippocampal GABAergic Interneurons NR1 KO mice • Blunted MK-801-induced hyperlocomotor activity in PV-specific NR1 KO mice • Hyperlocomotor activity in forebrain pyramidal cells-specific NR1 KO mice	• Reduced nest building, impaired social and sexual interactions in NR1 KD mice • Reduced sociability and sensitivity to NMDA-induced seizures in GRIN (D481N) mice • Disrupted nesting behavior in GRIN1 compound het mice • Social interaction deficits in CA3 NR1 KO mice • Anhedonia-like behavior in cortical and hippocampal GABAergic Interneurons NR1 KO mice • Mating and nest-building deficits in cortical and hippocampal GABAergic Interneurons NR1 KO mice • Anhedonia-like behavior in PV NR1 KD mice • Decreased self-care in forebrain pyramidal cells-specific NR1 KO mice	• Sensorimotor gating deficits in NR1 KD mice • Increased startle reactivity in GRIN1 (D481N) mice • Sensorimotor gating deficits in GRIN1 compound het mice • Sensorimotor gating deficits in cortical and hippocampal GABAergic Interneurons NR1 KO mice • Sensorimotor gating deficits in mPFC and sensory cortex pyramidal cells-specific NR1 KO mice	• Cognitive inflexibility, abnormal selective attention, and spatial cognitive deficits in NR1 KD mice • Deficits in spatial recognition, spatial reference learning and memory in GRIN (D481N) mice • Deficits in spatial learning in GRIN1 compound het mice • Deficits in process of pattern separation and spatial working memory deficits in DG NR1 KO mice • Impairments in spatial memory, deficits in nonspatial memory and trace fear conditioning in forebrain pyramidal cells-specific NR1 KO mice • Deficits in avoidance task learning and memory formation in CA1 NR1 KD mice • Deficits in associative memory recall in CA3 pyramidal cells NR1 KO mice • Learning impairments in CA3 NR1 KO mice • Deficits in spatial working memory in cortical and hippocampal GABAergic Interneurons NR1 KO mice • Deficits in spatial working, short- and long-term recognition memory in PV-specific NR1 KO mice • Deficits in working memory in PV-specific NR1 KO mice • Impairments in short-term memory in mPFC and sensory cortex pyramidal cells NR1 KO mice	• Increased axonal arborization in NR1 KD mice • Faster, premature developed projection neurons of the corpus callosum in NR1 KD mice • Altered dendritic differentiation, branching, and somatosensory pattern in NR1 KD mice • Reduced synapse number in an age-dependent manner in NR1 KD mice • Decreased NMDA currents, reduced 2-deoxyglucose uptake in the neocortex in NR1 KD mice • Increased amplitudes of auditory and visual ERPs in NR1 KD mice • Attenuated cortical and hippocampal theta-gamma phase coupling in NR1 KD mice • Increased dendritic length and reductions in 14-3-3epsilon and DISC1 protein expression in NR1 KD mice • Impaired hippocampal LTP in GRIN1 (D481N) mice • Striatal dopaminergic and serotonergic hyperfunction in GRIN1 compound het mice • Reduced firing rate from CA3 pyramidal cells during context-specific modulation in DG NR1 KO mice • Impaired LTP in DG NR1 KO mice • Lack of NMDA-mediated synaptic currents and LTP in forebrain pyramidal cells-specific NR1 KO mice • Impaired LTP at the C/A-CA3 synapse in CA3 pyramidal cells NR1 KO mice • Increased firing of cortical excitatory neurons with reduced neuronal synchrony in cortical and hippocampal GABAergic Interneurons NR1 KO mice • Increased gamma oscillation power and decreased theta oscillation power of LFP In hippocampus of PV-specific NR1 KO mice • Enhanced cortical gamma rhythm with impaired gamma rhythm induction after optogenetic drive of PV interneurons with decreased sensitivity to MK-801 on gamma oscillations and stereotypic behavior in PV-specific NR1 KO mice • Decreased D2R and GIRK2 expression in the forebrain from forebrain pyramidal cells-specific NR1 KO mice	Forrest et al., 1994; McHugh et al., 1996, 2007; Tsien et al., 1996; Iwasato et al., 1997; Mohn et al., 1999; Huerta et al., 2000; Kew et al., 2000; Rondi-Reig et al., 2001; Ballard et al., 2002; Cheli et al., 2002, 2006; Duncan et al., 2002, 2004; Nakazawa et al., 2002; Lee et al., 2005; Moy et al., 2006; Rajji et al., 2006; Bickel et al., 2007; Niewoehner et al., 2007; Labrie et al., 2008; Dzirasa et al., 2009; Halene et al., 2009; Belforte et al., 2010; Korotkova et al., 2010; Ramsey et al., 2011; Carlen et al., 2012; Rompala et al., 2013; Finlay et al., 2015; Tatard-Leitman et al., 2015; Bygrave et al., 2016
NR2A subunit knockout	• Hyperlocomotor activity in NR2A KO mice	• No changes observed	• No disruption in sensorimotor gating in NR2A KO mice	• Deficits in spatial learning, fear coding, and discrimination learning in NR2A KO mice	• Impairments in hippocampal LTP in NR2A KO mice • Reduced NMDAR EPSCSs and LTP in the CA3–CA1 synapse in hippocampal slices from NR2A KO mice	Carlsson and Lindqvist, 1963; Sakimura et al., 1995; Ito et al., 1997; Kiyama et al., 1998; Sprengel et al., 1998; Moriya et al., 2000; Brigman et al., 2008
NR2B subunit knockout	• Hyperlocomotor activity in CamKIIa NR2B KO mice	• No changes observed	• Enhanced startle responses to acoustic stimuli in NR2B het mice	• Impairments in spatial and nonspatial learning and memory in CamKIIa NR2B KO mice • Deficits in selective, short-term, spatial working memory in hippocampal pyramidal cells-specific NR2B KO mice	• Abolished LTD in the CA1 hippocampal neurons from NR2B KO mice • Diminished NMDAR EPSCs and LTP in the Fim-CA3 synapse from hippocampal slices of NR2B het mice • Decreased NMDAR-mediated EPSCs with accelerated decay kinetics and reduced cellular LTP in the hippocampus in hippocampal pyramidal cells-specific NR2B KO mice and CamKIIa NR2B KO mice	Kutsuwada et al., 1996; Ito et al., 1997; Takeuchi et al., 2001; von Engelhardt et al., 2008; Badanich et al., 2011
mGluR knockout	• Altered responses to stimulants in PV mGluR5 KO mice	• Increased compulsive-like behavior in PV-specific mGluR5 KO mice	• Abnormal sensorimotor gating in PV mGluR5 KO mice	• Memory impairments in PV mGluR5 KO mice	• Reduced numbers of PV interneuron density in PV mGluR5 KO mice • Decreased inhibitory currents in PV mGluR5 KO mice • Alterations in ERPs and brain oscillatory activity in PV mGluR5 KO mice • Deficient developmental switch from NR2B- to NR2A-containing receptors at synapses onto hippocampal CA1 pyramidal neurons and pyramidal primal visual cortical neurons in mGluR5 KO mice	Matta et al., 2011; Uzunova et al., 2014; Barnes et al., 2015
NRG1 and ErbB4 knockout	• Hyperlocomotor activity and increased motor coordination in NRG1 het mice • Hyperlocomotor activity in NRG1 het mice • Hyperlocomotor activity in ErbB4 het mice	• No changes observed	• Impairments in sensorimotor gating in NRG1het mice • Sensorimotor gating deficits in type III NRG1 het mice • Sensorimotor gating deficits in CNS-specific ErbB2 and ErbB4 dKO mice	• Impaired performance on delayed alternation task in type III NRG1 het mice • Disruption in cue utilization during Morris water maze in CNS-specific ErbB4 KO mice	• Deficits in subpopulations of cortical GABAergic interneurons in neuronal-specific ErbB4 het mice or type III NRG1 null mice • Enlarged lateral ventricles and decreased dendritic spines on hippocampal pyramidal neurons in NRG1 het mice • Reduced power of kainate-induced gamma oscillations in ErbB4 het mice • Reduced numbers of PV and CB interneuron density in the hippocampus and GABAergic interneurons in the cortex in ErbB4 het mice • Delayed postnatal motor development in CNS-specific ErbB4 KO mice • Decreased spine density in the cortex and hippocampus in CNS-specific ErbB2 and ErbB4 dKO mice • Thinning of myelin sheath of the corpus callosum, altered oligodendrocyte morphology, increased number of cells expressing differentiated oligodendrocytes, dopaminergic abnormalities in dominant negative ErbB4 mutant mice • Bath application of NRG1 reduces NMDAR-mediated currents in PFC pyramidal neurons • Increased expression of NRG1 and ErbB4 proteins upon chronic MK-801 administration in rats • NRG1-ErbB4 signaling may either suppress Src-mediated enhancement of synaptic NMDAR function or through upregulation of EAAC1	Gassmann et al., 1995; Erickson et al., 1997; Gerlai et al., 2000; Stefansson et al., 2002; Flames et al., 2004; Golub et al., 2004; Thuret et al., 2004; Gu et al., 2005; Roy et al., 2007; Ago et al., 2008; Chen et al., 2008; Barros et al., 2009; Fisahn et al., 2009; Feng et al., 2010; Pitcher et al., 2011; Yu et al., 2015
14-3-3 protein knockout	• Hyperlocomotor activity in 14-3-3 FKO mice • Hyperlocomotor activity in 14-3-3ζ KO mice • Enhanced anxiety-like behavior in 14-3-3ε het mice	• Deficits in social interaction in 14-3-3 FKO mice	• Deficits in sensorimotor gating in 14-3-3 FKO mice • Reduced sensorimotor gating in 14-3-3ζ KO mice	• Deficits in working memory and associative learning and memory in 14-3-3 FKO mice • Cognitive deficits in 14-3-3ζ KO mice • Weak defects in working memory in 14-3-3ε het mice	• Reduction in the NMDAR-mediated synaptic currents in CA1 pyramidal neurons in 14-3-3 FKO mice • Protein levels of NR1 and NR2A subunits are reduced in the PSD fraction of 14-3-3 FKO mice • Impaired LTP at hippocampal CA3-CA1 synapses in 14-3-3 FKO mice • Altered synaptic connectivity in the prefrontal cortical synapses in 14-3-3 FKO mice • Altered synaptic neurotransmission and plasticity in 14-3-3ζ KO mice • Abnormal neuronal migration and axonal guidance defects in the hippocampus in 14-3-3ζ KO mice • Increased levels of DA in striatum in 14-3-3ζ KO mice	Ikeda et al., 2008; Cheah et al., 2012; Ramshaw et al., 2013; Qiao et al., 2014; Foote et al., 2015; Xu et al., 2015

### Knockout of NR2A or NR2B Subunits

Amongst four NR2 subunits (A–D), the NR2A and NR2B subunits are known to predominantly function in the forebrain. They impart different characteristics on functional NMDARs, with NR2A-containing NMDARs having more rapid kinetics than NR2B-containing NMDARs (Kutsuwada et al., 1992; Monyer et al., 1994; Loftis and Janowsky, 2003). A switch of NR2B to NR2A subunit expression in the forebrain and sensory systems is linked with the timing of critical period for sensory plasticity, making NR2B and NR2A subunits particularly known for its involvement in postnatal brain development (Monyer et al., 1994; Sheng et al., 1994; Yashiro and Philpot, 2008). At birth, NR2B is the predominant subunit, whereas NR2A subunit expression begins around postnatal day 3 (P3), after which there is a gradual increase expression of NR2A subunits (Monyer et al., 1994; Sheng et al., 1994; Zhong et al., 1995). Furthermore, NR2A and NR2B subunits are known to interact differentially with binding partners in the postsynaptic density (PSD) and may potentially activate different downstream signaling pathways according to changes in NMDAR subunit composition during development (Massey et al., 2004). Thus, various genetic animal models with the loss of NR2A or NR2B subunits have been studied to understand how expression and function of these subunits may contribute to NMDAR hypofunctionality.

As suggested by its early expression in development, homozygous NR2B KO mice are perinatally lethal. These mice were examined shortly after birth in hippocampal slices. The loss of NR2B abolishes synaptic NMDA responses and long term depression (LTD) in the CA1 hippocampal neurons, suggesting that NR2B expression has an essential role in synaptic plasticity (Kutsuwada et al., 1996). Since homozygous NR2B KO mice died perinatally, the heterozygous NR2B mutant mice were generated to study the effect of NR2B deletion in adulthood. One study found diminished NMDAR excitatory postsynaptic currents (EPSCs) and LTP in the fimbrial (Fim)-CA3 synapse when recorded from hippocampal slices of the heterozygous NR2B mutant mice (Ito et al., 1997). A separate study reported that these mice exhibit enhanced startle responses to acoustic stimuli (Takeuchi et al., 2001).

Mouse models with deletion of NR2B subunits in certain brain regions and/or cell types were further studied to understand the role of NR2B subunits in cognitive functions. A forebrain pyramidal cells-specific NR2B KO mice were created by genetic deletion of NR2B beginning at early postnatal development using the calcium^2+^/calmodulin-dependent protein kinase II-alpha (CamKII-α) promoter. These mice display hyperlocomotor activity and exaggerated depressant effect (Badanich et al., 2011). Similarly, another study done on these forebrain pyramidal cells-specific NR2B KO mice reports impairment in spatial and non-spatial learning and memory, whereas hippocampal pyramidal cells-specific NR2B KO mice only display selective, spatial working memory deficits. Nevertheless, both forebrain and hippocampal pyramidal cells-specific NR2B KO mice exhibit decreased NMDAR-mediated EPSCs with accelerated decay kinetics and reduced cellular LTP in the hippocampus (von Engelhardt et al., 2008).

In contrast to NR2B KO mice, NR2A KO mice are viable. They exhibit hyperlocomotor activity and cognitive dysfunctions without any sensorimotor gating deficits (Sprengel et al., 1998; Brigman et al., 2008). The cognitive deficits exhibited in NR2A KO mice include spatial learning, fear coding, and discrimination learning impairments accompanied by impaired hippocampal LTP. When recorded from hippocampal slices generated from the NR2A KO mice, there was reduced NMDAR-mediated EPSCs and LTP in the CA3-CA1 synapse (Ito et al., 1997). Overall, the changes displayed in both genetic mutants of NR2A and NR2B subunits highlight the crucial role that both subunits play in synaptic plasticity and cognitive functions (Sakimura et al., 1995; Kiyama et al., 1998; Moriya et al., 2000). Due to overlapping roles of NR2A and NR2B subunits in learning and memory, NR2A and NR2B mutant mice would serve as great models to study the pathophysiology of cognitive symptoms of schizophrenia (Table 4).

### Overexpression or Re-expression of NMDAR Subunits

Multiple studies have also examined behavioral and molecular consequences upon overexpressing different NMDAR subunit(s). In one study, overexpression of the NR1 subunit into the hippocampus of wild type mice via AAV-mediated delivery led to increased fear memory and neurogenesis as well as delayed onset of severe seizures (Kalev-Zylinska et al., 2009). Other studies reported that overexpression of NR2B subunits in the forebrain of a wild type mice improve learning and memory and enhance NMDAR-dependent synaptic potentiation (Tang et al., 1999, 2001). In addition, transgenic mice with forebrain pyramidal cells-specific overexpression of NR2B postnatally exhibit enhanced social recognition memory for different strains and animal species (Jacobs and Tsien, 2012). Another group found that overexpression of NR2B subunit in aged wild type mice enhances long-term spatial memory. On the other hand, transgenic mice with forebrain-specific NR2A overexpression display long-term memory deficits, suggesting that subtle shifts in subunit compositions can have major effects on native receptor function and highlights how different subunit compositions can produce receptors with different functional properties (Madden, 2002; Cui et al., 2013).

Behavioral deficits in genetic mutants of NMDAR subunit(s) could also be rescued by restoring NMDAR subunit expression levels. In the NR1-KD mice, the hypomorphic insertion mutation could be excised in the Cre recombinase-dependent manner to restore NR1 in a temporal-regulated fashion. This allowed to investigate behavioral phenotypic rescue at different stages of development upon global restoration of NMDARs (Mohn et al., 1999; Mielnik et al., 2017). The mice with inducible NR1 rescue during adolescence or in adulthood achieved similar levels of functional recovery in some of cognitive deficits and negative symptoms-related behavioral changes, with cortically-mediated behaviors completely or nearly rescued. However, subcortically-mediated behaviors, such as hyperlocomotor activity and anxiety-related behaviors were only partially rescued. This suggested that higher-order brain functions are potentially more amenable to treatment in adulthood and unencumbered by critical period (Mielnik et al., 2017). Overexpressing NR2B in aged wild type mice was able to rescue age-associated impairments in hippocampal-dependent spatial memory. This was accompanied by enhanced LTP and increased NMDAR-mediated EPSPs from hippocampal slices generated from these aged mice with increased NR2B subunit expression, suggesting that increasing NR2B expression in aged animals can also enhance memory and synaptic transmission (Brim et al., 2013). These studies further point to how re-expression or overexpression of NMDAR subunits rescue behavior deficits associated to symptoms of schizophrenia, suggesting that enhancing levels of NMDAR subunits may ameliorate NMDAR hypofunctionality. However, limitations in fully restoring NMDAR subunits or rescuing behavioral phenotypes associated with subcortically-mediated behaviors still remain to be a challenge and may require further studies.

Moreover, the NMDAR levels can be influenced by activity or experience. Multiple studies showed that both NR1 and NR2A subunit protein expressions increase in the hippocampus of 1, 2, and 3-month old rats following habituation to a new environment via open field test and following novel object recognition task (Baez et al., 2013; Cercato et al., 2016). Shanmugasundaram et al. reported that 6 h after training rats in a radial maze along 10 consecutive days, there is an increase in NR1 and NR2B protein levels in synaptosomal fractions from the hippocampus and an increase in synaptic NR1 and NR2A levels in the PFC (Shanmugasundaram et al., 2015). In conclusion, NMDAR levels are influenced by genetic overexpression, re-expression of NMDAR subunit(s) and are also experience- and activity-dependent.

### Metabotropic Glutamate Receptors (mGluRs)

Glutamatergic neurotransmission is mediated by both metabotropic and ionotropic glutamate receptors. While ionotropic glutamate receptors such as NMDA and quisqualate/a-amino-3-hydroxy-5-methyl-5-isoxazolepropionic acid (AMPA) receptors are responsible for fast excitatory transmission, metabotropic receptors have a modulatory role (Kew and Kemp, 2005). Metabotropic glutamate receptors (mGluRs) are further subdivided into three groups, with Group I metabotropic glutamate receptors (mGluR1 and mGluR5) mainly localized postsynaptically and positively linked to phospholipase C (PLC), which is known to potentiate glutamate function at NMDARs. In contrast, Group II (mGluR2 and mGluR3) and III (mGluR4, mGluR6, mGluR7, and mGluR8) metabotropic glutamate receptors are primarily presynaptic and are negatively linked to adenylyl cyclase (AC), which is known for its role in modulating neurotransmitter release (Javitt, 2004). Moreover, Group I mGluRs induce the enhancement of NMDAR currents and are involved in the direct phosphorylation of the NMDARs (Cartmell et al., 2000; Pisani et al., 2001). In particular, mGluR5s at the PSDs are physically linked to NMDARs and are known to enhance NMDAR function, this makes mGluRs a leading target for novel therapeutics to treat cognitive symptoms of schizophrenia (Ehlers, 2002; Yang et al., 2004; Gray et al., 2009).

Animal model studies have reported that the excitatory glutamatergic neurotransmission through ionotropic and metabotropic glutamate receptors is necessary for the correct postnatal development of the GABAergic network, with mGluR5s having a fundamental role in the development of PV interneurons (Uzunova et al., 2014; Barnes et al., 2015). PV-specific mGluR5 KO mice display memory impairments, increased compulsive-like behaviors, abnormal sensorimotor gating, and altered responsiveness to stimulants. These behavioral changes are accompanied by reduced PV interneuron density, decreased inhibitory synaptic currents, as well as abnormal ERPs and brain oscillatory activity (Barnes et al., 2015) Another study showed that mGluR5 KO mice exhibit deficient developmental switch from NR2B- to NR2A-containing receptors at synapses onto hippocampal CA1 pyramidal neurons and pyramidal primary visual cortical neurons (Matta et al., 2011), suggesting that mGluR5 plays an important role in regulating age-dependent expression of NMDAR subunits.

In addition, pharmacological modulations of different mGluR subtypes may provide alternative mechanisms to normalize aberrant neurotransmission induced by NMDAR hypofunction (Moghaddam et al., 1997; Marino and Conn, 2002). A licensed drug originally discovered for the prevention of relapse in alcohol dependence, acamprosate, interferes with mGluR5-dependent glutamate release. Preclinical studies with acamprosate suggest that it normalizes glutamate release and NMDAR functions without altering the normal glutamatergic neurotransmission (De Witte et al., 2005). Other approaches have also been made to normalize glutamatergic neurotransmission by using glutamate release inhibitors, such as selective mGluR2/3 agonists (LY2140023), which alleviates positive and negative symptoms in schizophrenia patients during phase II clinical trials, but failed in phase III clinical trials (Patil et al., 2007; Li et al., 2015). Thus, far, mGluR-targeting compounds have yet to be clinically approved for schizophrenia patients (Moghaddam and Adams, 1998; Kinon et al., 2011; Hopkins, 2013). Further studies in different animal models may facilitate this process by establishing functional link between mGluRs and NMDAR hypofunctionality (Table 4).

### Neuregulin 1 (NRG1) and ErbB4 Receptor

The neuregulins (NRGs) are a family of growth and differentiation factors encoded by four genes (NRG1-4). The NRGs bind to the ErbB family of tyrosine kinase transmembrane receptors (ErbB1-4). Amongst these receptors, ErbB4 is likely to be the major mediator of NRG1 functions in the brain (Mei and Xiong, 2008). Both NRG1 and ErbB4 receptor have been identified as candidate risk genes for schizophrenia through genetic studies (Stefansson et al., 2002, 2003; Yang et al., 2003; Corvin et al., 2004; Zhao et al., 2004; Owen et al., 2005; Hahn et al., 2006). Accumulating evidence supports that NRG1 and ErbB4 play crucial roles in neurodevelopment and in the modulation of NMDAR signaling (Ozaki et al., 1997; Rieff et al., 1999; Anton et al., 2004). NRG1 is expressed in the PFC, hippocampus, cerebellum, and substantia nigra, where it regulates the expression and function of NMDA, γ-aminobutyric acid (GABA), and acetylcholine receptors in both humans and in rodents (Kerber et al., 2003; Corfas et al., 2004; Law et al., 2004; O'Tuathaigh et al., 2007). ErbB4 receptor is involved in neurogenesis, synaptic plasticity, neuronal migration, synapse formation and the regulation of NMDAR-mediated neurotransmission (Rieff et al., 1999; Anton et al., 2004; Flames et al., 2004; Ghashghaei et al., 2006; Li et al., 2007).

In animal model studies, the NRG1 homozygous null mutant mice are found to be embryonically lethal (Erickson et al., 1997). This led to the studies of viable, NRG1 heterozygous null mutant mice. These mice exhibit hyperlocomotor activity and spatial learning impairments (Gerlai et al., 2000). Another NRG1 hypomorphic mice were created by deleting the transmembrane domain of the NRG1 protein. These heterozygous mice exhibit hyperlocomotor activity and impaired sensorimotor gating, which was accompanied by decreased MK-801 binding from the forebrain, suggesting fewer functional NMDARs (Stefansson et al., 2002). Subsequently, studies using mutant ErbB4 or NRG1 mouse lines revealed that the NRG1-ErbB signaling is crucial for tangential migration of cortical GABAergic interneurons, as the number of GABAergic interneurons in the embryonic cortex is decreased in either the neuronal specific NRG1 KO mice or a line of ErbB4 null mice (Flames et al., 2004). Another NRG1 heterozygous mice were generated by targeted disruption of type III NRG1 (Chen et al., 2008), which is one of many NRG1 isoforms highly expressed throughout embryonic and postnatal brain development and are implicated in schizophrenia (Meyer et al., 1997; Anton et al., 2004; Longart et al., 2004). These NRG1 heterozygous mice exhibit impaired performance on delayed alternation task and sensorimotor gating deficits. They also display enlarged lateral ventricles and decreased dendritic spine density on hippocampal pyramidal neurons (Chen et al., 2008).

The ErbB4 KO mice are also embryonically lethal (Gassmann et al., 1995). Thus, the heterozygous ErbB4 mutant mice were most widely used to study animal models of ErbB4 receptor. These mice mainly exhibit hyperlocomotor activity and reduced power of kainate-induced gamma oscillations, accompanied by reduced numbers of PV interneuron density in the hippocampus as well as reduced calbindin (CB) and GABAergic interneurons in the cortex (Stefansson et al., 2002; Flames et al., 2004; Fisahn et al., 2009). Other animal models with ErbB4 deletion in brain region and/or cell-type-specific manner have also been studied. Central nervous system (CNS)-specific ErbB4 KO mice exhibit delayed postnatal motor development, and disrupted cue utilization during Morris water maze (Golub et al., 2004). CNS-specific double KO (dKO) mice lacking both ErbB2 and ErbB4 at early embryonic stages display sensorimotor gating deficits, which is accompanied by decreased spine density in the cortex and hippocampus (Barros et al., 2009). Interestingly, another CNS-specific ErbB4 homozygous null mice did not exhibit any motor function impairments or any other behavioral deficits (Thuret et al., 2004). Moreover, expression of dominant negative ErbB4 in oligodendrocytes and myelination Schwann cells from E15 result in thinning of the myelin sheath of the corpus callosum, altered oligodendrocyte morphology, increased number of cells expressing differentiated oligodendrocytes as well as dopaminergic abnormalities, which is predicted to result from defective myelin (Roy et al., 2007; Table 4).

It is worth noting that most studies seem to suggest a link between increased NRG1-ErbB signaling and decreased NMDAR function in the PFC. Bath application of NRG1 reduces NMDAR-mediated currents in PFC pyramidal neurons (Gu et al., 2005). A postmortem tissue-stimulation study also reported that NRG1 stimulation suppresses NMDAR activation in the human PFC (Hahn et al., 2006). Conversely, blockade of NMDARs via chronic MK-801 administration in rats increases the expression of NRG1 and ErbB4 proteins (Feng et al., 2010). Moreover, others have found that NRG1-ErbB4 signaling may cause NMDAR hypofunction by either suppressing Src-mediated enhancement of synaptic NMDAR function or through upregulation of excitatory amino-acid carrier 1 (EAAC1) (Pitcher et al., 2011; Yu et al., 2015). Given that most of the animal models reviewed here were based on deficiency of the NRG1-ErbB4 signaling, caution must be exercised in interpreting these results in the context of NMDAR hypofunctionality.

### 14-3-3 Proteins

14-3-3 refers to a family of brain-enriched proteins (β, ε, η, γ, σ, θ, and ζ isoforms) encoded by seven genetic loci (YWHAB, YWHAE, YWHAH, YWHAG, YWHAS, YWHAQ, and YWHAZ) (Altar et al., 2009). 14-3-3 proteins exist as homo- or heterodimers and bind to target proteins via specific phosphoserine/phosphothreonine-containing motifs. By interacting with its target proteins, 14-3-3 modulates a wide range of cellular processes (Muslin et al., 1996; Berg et al., 2003). In the nervous system, 14-3-3 proteins are enriched at synapses and regulate synaptic transmission and plasticity (Martin et al., 1994; Broadie et al., 1997). Based on previous studies, 14-3-3 proteins play a crucial role in surface expression of NR2C subunit in cerebellar granule cells (Chen and Roche, 2009), and 14-3-3 family members are found to associate and colocalize with NMDARs (Taya et al., 2007).

We have generated the 14-3-3 functional KO (FKO) mice by transgenic expression of a dimeric fourteen-three-three peptide inhibitor (difopein), which antagonizes the binding of 14-3-3 proteins to their endogenous partners in an isoform-independent manner to disrupt 14-3-3 functions in the brain (Wang et al., 1999; Masters and Fu, 2001; Cao et al., 2010; Foote et al., 2015). Based on our behavioral analyses, 14-3-3 FKO mice exhibit a full range of endophenotypes associated with core symptoms of schizophrenia. These behavioral changes include hyperlocomotor activity, social interaction impairments, deficits in sensorimotor gating, working memory, and associative learning and memory. In 14-3-3 FKO mice, we also identified a significant reduction in the NMDAR-mediated synaptic currents in CA1 pyramidal neurons. The protein levels of NMDA receptors, particularly NR1 and NR2A subunits, are selectively reduced in the PSD fraction of 14-3-3 FKO mice. These observations thus provide *in vivo* evidence linking 14-3-3 dysfunction to NMDAR hypofunction in forebrain neurons (Qiao et al., 2014; Foote et al., 2015). In a recent study, we reported that AAV-mediated expression of the 14-3-3 inhibitor specifically within the hippocampus alone is sufficient to induce several behavioral deficits including hyperactivity, impaired associative learning and memory, and reduced sensorimotor gating. Conversely, selectively restoring the function of 14-3-3 in the forebrain of the 14-3-3 FKO mice attenuate hyperlocomotor activity of the 14-3-3 FKO mice. In addition, we show that postsynaptic NMDA receptor levels are regulated by acute 14-3-3 manipulations (Graham et al., 2019). Taken together, findings from these animal model studies directly link 14-3-3 inhibition and NMDAR hypofunction in specific forebrain regions to certain schizophrenia-associated behavioral deficits.

Previous genetic and postmortem studies have identified 14-3-3ζ (*YWHAZ*) and 14-3-3ε (*YWHAE*) as candidate risk genes for schizophrenia (Middleton et al., 2005; Wong et al., 2005; Ikeda et al., 2008). This led to animal model studies to determine whether the loss of 14-3-3ζ or 14-3-3ε isoform leads to behavioral changes related to symptoms of schizophrenia. The 14-3-3ζ KO mice display hyperlocomotor activity, sensorimotor gating and cognitive deficits. These mice also display alterations in synaptic neurotransmission and plasticity, abnormal neuronal migration and axonal guidance defects in the hippocampus, and increased levels of DA in the striatum (Cheah et al., 2012; Ramshaw et al., 2013; Xu et al., 2015). As 14-3-3ε KO mice are found to be neonatally lethal, 14-3-3ε heterozygous mice are studied for behavioral deficits related to schizophrenia. 14-3-3ε heterozygous mice display weak defects in working memory and enhanced anxiety-like behavior (Ikeda et al., 2008; Toyo-oka et al., 2014). Taken together, these results suggest a link between these 14-3-3 isoforms to schizophrenia. Further studies are required to understand whether NMDAR hypofunctionality may be mediating behavioral deficits associated with schizophrenia exhibited in 14-3-3 isoform-specific animal models (Table 4).

## Conclusion

Here we reviewed various animal models of NMDAR hypofunctionality created through pharmacological and genetic approaches. Animal models of schizophrenia have also been generated by using dopaminergic agonists, such as amphetamine and apomorphine, which primarily induce behavioral changes limited to hyperlocomotor activity, stereotypy, and sensorimotor gating deficits (Kokkinidis and Anisman, 1980; Sharp et al., 1987; Swerdlow et al., 1994; Swerdlow and Geyer, 1998; Marcotte et al., 2001; Jones et al., 2011). Similarly, direct infusion of GABA_A_ receptor antagonist picrotoxin into the mPFC only causes sensorimotor gating deficits in rats (Japha and Koch, 1999; Marcotte et al., 2001). In comparison, as reviewed in this article, NMDAR antagonists induce a full range of behaviors corresponding to symptoms of schizophrenia, with robust changes in negative and cognitive symptoms-related behaviors. Moreover, some behavioral deficits induced by NMDAR antagonists are reversed by administration of APDs, including haloperidol and clozapine (Irifune et al., 1991; Noda et al., 1995; Verma and Moghaddam, 1996; Sams-Dodd, 1999; Abdul-Monim et al., 2006; Szlachta et al., 2017). Thus, administration of NMDAR antagonists provides a relatively valid animal model for studying symptomologies and pathophysiology of schizophrenia.

However, there are limitations in the use of NMDAR antagonists to model NMDAR hypofunctionality. First, we must ask whether pharmacologically induced alterations in the brain are a true representation of dysfunctional neural circuitry in schizophrenic patients. Olney et al. argued that since NMDAR antagonists cause NMDAR hypofunctionality throughout the brain, these animal models cannot target NMDARs in a neural circuit specific manner, thus may fail to precisely recapitulate schizophrenia pathophysiology (Olney et al., 1999). Second, schizophrenia is considered a neurodevelopmental disorder with deficits occurring during early brain development, yet a majority of animal studies have been focused on NMDAR antagonist-induced changes in adulthood (Harrison and Weinberger, 2005; Rapoport et al., 2005; Fatemi and Folsom, 2009; Powell, 2010). Further studies should be extended to address schizophrenia as a neurodevelopmental disorder by administering NMDAR antagonists at either prenatal or postnatal time period and examine their impacts on behavior and neural circuitry during development. Third, it is debatable whether some of the drug-induced behavioral changes in rodents are direct or even true correspondence of symptoms exhibited by schizophrenia patients. For example, hyperlocomotor activity measured in the open field test may have low relevance to psychosis in human patients (Powell and Miyakawa, 2006; Moore, 2010). This should contribute to limitations in interpretation of results obtained from these animal models with currently used behavioral paradigms (Moore, 2010). Fourth, behavioral and neurophysiological phenotypes exhibited in animal models of NMDAR antagonists are not specifically corresponding to symptoms of schizophrenia. For example, subchronic ketamine treatment in rodents leads to increased aggression, which is not a schizophrenia-related behavioral phenotype (Becker et al., 2003). Other behavioral phenotypes in pharmacological models, such as social interaction deficits, hyper responsiveness to sensory stimuli as well as cognitive deficits, overlap with symptoms associated with autism spectrum disorder (ASD) (Crawley, 1999, 2012). Finally, NMDAR antagonists cause neurotoxicity, evidenced by neuronal vacuolization, neuronal necrosis, and other cytotoxic changes. This must be taken into consideration when interpreting results from those animal studies, especially for chronic models of NMDAR antagonist (Olney et al., 1989, 1991; Nakao et al., 2003). Neurotoxicity should also be considered by the researcher when deciding on the dosage, regimen, and the type of NMDAR antagonist proposed to be used depending on the aim of the study.

Compared to pharmacological models, genetic animal models have several advantages for studying NMDAR hypofunctionality in relation to schizophrenia. First, schizophrenia is a disease of common symptomatology with etiological heterogeneity caused by various genetic and environmental factors (Takahashi, 2013). Human genome wide association studies (GWAS) have identified NMDAR genes or genes encoding proteins that regulate NMDARs to be candidate risk genes for schizophrenia (Fromer et al., 2014; Purcell et al., 2014; Schizophrenia Working Group of the Psychiatric Genomics Consortium, 2014; Harrison, 2015). There is also evidence of reduced expression of some NMDAR-subunits in schizophrenia patients (Balu, 2016). Thus, genetic animal models of NMDAR hypofunction may have certain construct validity for schizophrenia. However, we must emphasize that NMDAR hypofunction itself does not guarantee the animal model of schizophrenia, since NMDAR hypofunction leads to different neurological and psychiatric conditions, which do not necessarily correspond to the symptoms of schizophrenia. Second, *de novo* mutations in schizophrenia patients are overrepresented in glutamatergic postsynaptic proteins comprising of NMDAR complexes, making genetic animal models particularly useful in dissecting the signaling pathways and molecular mechanisms leading to NMDAR hypofunction (Forrest et al., 1994; Fromer et al., 2014). As reviewed here, some of the genetic animal studies, such as NRG1/ErbB4 and 14-3-3 animal models, provided evidence to support NMDAR hypofunctionality as a potential convergence point for symptoms of schizophrenia (Snyder and Gao, 2013). Third, various genetic models have been created to either directly or indirectly downregulate NMDAR functions in a spatial and temporal controlled manner. Some of these models specifically examine the role of NMDAR functions in different cell types and/or brain regions, while others have studied the impact of NMDAR hypofunction at different neurodevelopment stages. These studies may help reveal “how” genetic and environmental factors (signaling pathways) lead to NMDAR hypofunctionality and behavioral deficits, as well as “where” (in which neural circuits) and ‘when' these changes occur during neurodevelopment. Such information will be crucial for our understanding of the disease progress. In the future, some of the animal models may provide us useful platforms for identifying novel therapeutics for schizophrenia patients.

## Author Contributions

GL was responsible for drafting, writing, reviewing, and editing this manuscript. YZ was responsible for writing, reviewing, and editing the manuscript with suggestive ideas on formatting and outlining this manuscript.

### Conflict of Interest Statement

The authors declare that the research was conducted in the absence of any commercial or financial relationships that could be construed as a potential conflict of interest.
